# A Case of Arsenical Poisoning Treated with Dialysed Iron

**Published:** 1878-01-20

**Authors:** Thomas B. Reed


### *A CASE OF ARSENICAL POISONING TREATED WITH DIALYZED IRON.*

By Thomas B. Reed, M. D.

A case of arsenical poisoning occurred lately in my private practice, which seems to be valuable enough for publication, both on account of the completeness of the details and the intelligence and reliability of the patient, but especially as it is, so far as I am aware, the first case where the new remedy of "dialyzed iron" has been put to the test as an antidote.

As I was leaving my office one morning, a few weeks ago, a young lady patient, Miss S——, hastily entered, with a face indicative of intense pain and nervous disturbance, saying, "Doctor, I am poisoned." Her story was as follows: While attending to the wants of a valuable servant, who was sick and confined to her bed, Miss S. found, hidden away in the servant's trunk, a paper of arsenious acid, which had been procured by Mrs. S., some weeks before, foi use as a poison for rats. As this servant had been in ill health for sometime, and morbid and melancholy, Miss S. at once very naturally, and no doubt very rightly, supposed that she had secreted the poison for the purpose of taking her own life. Quietly placing the packet of arsenic (which was open) in her pocket, she continued her duties, intending, at the earliest moment, to put it in a safe place. Days elapsed, the arsenic was forgotten, stored away in the pocket of her wrapper, until this unlucky morning, when, putting a couple of handsful of gum-drops and bon-bons into her arsenic pocket, she sat down to her sewing machine and her confectionery. She noticed, from time to time, as she sewed, more powder upon the drops than seemed usual, but she continued quietly to dust them off as she ate, and went on with her work. Can anything be more absurdly tragic than this unconscious suicide, deliberately eating gum-drops powdered with arsenic? Probably an hour and a half passed in this innocent amusement, when suddenly "becoming deathly sick, instantly followed by intense pain," as if, as she quaintly expressed it, "she had had a pure mustard plaster on the inside of her stomach," she was roused to the consciousness that some strange mischief was at work. Terrified on remembering the arsenic, she attempted, unsuccessfully, to relieve her stomach with warm water; then, unwilling to alarm her mother, who was also an invalid, she hastily threw on her street dress and hat, and hurried to my office, about two blocks away. Fortunately for both of us, I had upon my table a sample bottle of dialyzed iron (John Wyeth & Brother), and as soon as she told me she had taken arsenic, and before she began her story, I administered a half tablespoonful of the iron, well diluted in a tumbler of water. This gave her almost instant relief. I repeated the dose in ten minutes, and then gave her a bottle of the iron, directing her to take a similar dose every half hour, and, later, every hour during the day. I saw her at her home in a few hours after, but she had had no return of her pain, except some slight cramp in the lower bowel and limbs, and a dose of magnesia at night, with mucilaginous drinks, soft food, with occasional doses of the iron well diluted, kept up for a few days, completed her cure. At my request, the day after her attack, Miss S. put into my hands the pocket, cut from the wrapper, which she could not be persuaded to touch after her poisoning. This I transferred to a reliable analytical chemist, from whose report of his examination, now in my possession, I condense the following:

"In the pocket of a chintz dress, I found a small packet, labeled 'Arsenic—Poison,' and in this packet, a second envelope, open on its long and upper side, containing a white powder. Both outer and inner envelopes were worn, as letters carried in pockets are. Between the outer and inner envelopes was a white powder, and in the pocket itself, mixed with the powder, I found two sugar-crystallized, soft gum-drops, and one sugar-coated bon-bon, all three richly covered with the powder. The powder, which with a brush I took away from the gum-drops, and the dragee, weighed $3\frac{1}{2}$ grains, and the remaining powder, after separating the gum and sugar, weighed 2 1-16 grains. In the pocket, I found also $6\frac{5}{8}$ grains of the white powder. The powder obtained from the gum-drops and dragee gave all the tests arsenious acid gives."

What amount of arsenious acid my patient swallowed, it is, of course, impossible to say. It is certain that, from this open package of arsenic, a considerable quantity escaped into the pocket, and the gum-drops were mixed with it, as she states "that she had to dust the powder off upon her work as she ate," and the three remaining after show 2 1-16 grains of arsenious acid upon them on examination by the chemist. I have, perhaps, been unnecessarily full in the details of this case, but I think they have established several facts: 1. That my patient did swallow, in the space of an hour or more, numerous poisonous doses of arsenious acid in powder; 2. That I found her with most marked symptoms of arsenical poisoning; and, 3. That by the administration of moderate doses of dialyzed iron, well diluted, I was enabled to give her immediate and certain relief, and ultimate and entire restoration to health. I do not propose, in the limits of this paper, to discuss the exact chemistry of the dialyzed iron. It is, I believe—when properly prepared, as I have since investigated carefully the process of its formation—a solution of peroxide of iron in the colloid form, with, perhaps, a trace of hydrochloric acid; but that it will, when very largely diluted with water, perfectly coagulate arsenious acid in solution, any one can satisfy himself in a five minutes' test. The only remaining point of interest, professionally, is, will it neutralize arsenious acid when taken in powder (bulk) into the stomach? It is held by most authorities, I believe, that when arsenious acid is taken in bulk into the stomach, the iron antidote is not reliable. (See Dunglison, R. J., latest paper on the subject, in his "Practitioner's Reference Book," page 229.) Yet, we know, from daily experience, that arsenious acid is absorbed by the stomach when taken in minute doses, and I think the evidence in this case shows that arsenic in powder did poison when presented to and acted upon by a comparatively empty stomach (at least three hours having elapsed since her breakfast), and that the solution of peroxide of iron (dialyzed iron) did prove a prompt and reliable antidote, coagulating and neutralizing the arsenic. Arsenious acid acts as it is dissolved, and the antidote, if supplied, combines, *pari passu*, with the solution formed by the liquids of the stomach, and renders it inert before damage is done to the mucous coat of the stomach, or it is absorbed into the system. Within twenty seconds after I learned that arsenic had been swallowed, I sent a full dose of the antidote after the poison, and with positive and immediate relief to the patient. My experience with dialyzed iron, as a pleasant and efficient means of introducing iron into the economy, is too limited for an opinion, but I feel disposed, from the history of this case, to strongly recommend it as a safe, reliable, and always-ready-at-a-moment's-notice remedy, and antidote for arsenical poisoning.

